# Music Community, Improvisation, and Social Technologies in COVID-Era Música Huasteca

**DOI:** 10.3389/fpsyg.2021.648010

**Published:** 2021-05-31

**Authors:** Daniel S. Margolies, J. A. Strub

**Affiliations:** ^1^History Department, Virginia Wesleyan University, Virginia Beach, VA, United States; ^2^Butler School of Music, University of Texas-Austin, Austin, TX, United States

**Keywords:** son huasteco, coronavirus, music, improvisation, livestream, youtube, huapango, digital intimacy

## Abstract

This article examines two interrelated aspects of Mexican regional music response to the coronavirus crisis in the música huasteca community: the growth of interactive huapango livestreams as a preexisting but newly significant space for informal community gathering and cultural participation at the onset of the coronavirus pandemic, and the composition of original verses by son huasteco performers addressing the pandemic. Both the livestreams and the newly created coronavirus disease (COVID) verses reflect critical improvisatory approaches to the pandemic in música huasteca. The interactive livestreams signaled an *ad hoc* community infrastructure facilitated by social media and an emerging community space fostered by Do-It-Yourself (DIY) activists. Improvised COVID-related verses presented resonant local and regional themes as a community response to a global crisis. Digital ethnography conducted since March 2020 revealed a regional burst of musical creativity coupled with DIY intentionality, a leveling of access to virtual community spaces, and enhanced digital intimacies established across a wide cultural diaspora in Mexico and the USA. These responses were musically, poetically, and organizationally improvisational, as was the overall outpouring of the son huasteco music inspired by the coronavirus outbreak. Son huasteco is a folk music tradition from the Huasteca, a geo-cultural region spanning the intersection of six states in central Mexico. This study examines a selection of musical responses by discussing improvisational examples in both Spanish and the indigenous language Nahuatl, and in the virtual musical communities of the Huasteca migrant diaspora in digital events such as “Encuentro Virtual de Tríos Huastecos,” the “Huapangos Sin Fronteras” festival and competition, and in the nightly gatherings on social media platforms developed during the pandemic to sustain the Huastecan cultural expression. These phenomena have served as vibrant points of transnational connection and identity in a time where physical gatherings were untenable.

## Introduction

In the middle of April 2020, as the coronavirus pandemic took hold globally in its initial wave, a trío huasteco called Los Yolpakis from Ixcatepec, Veracruz, Mexico released a new version of a tune from the regional vernacular repertory entitled “La Muerte” (Death). Applying a strong tradition of situational improvisation to reflect on an unprecedented historical moment, Los Yolpakis included original verses written expressly to respond to the pandemic. After introducing themselves in an enthusiastic unison—a style common among son huasteco video performances—the trío sang verses in both Spanish and the indigenous language Nahuatl (the most spoken indigenous language in Mexico), delivering a timely message of warning and concern. In both languages, they sang the huapango's standard first line: “Death is looking for me/To take me away” (“La muerte me anda buscando/para poderme llevar”). A common verse in “La Muerte,” found on popular recordings by foundational trío Armonia Huasteca, personifies death as showing up at a fandango—a communal dance—where the singer knows he will be easy to find, and where he “will play a tune that will move death incarnate to dance” (“pero como ando en fandango, muy fácil me va a encontrar/voy a tocarle un huapango para verla zapatear”) (Armonia Huasteca - La Muerte, 2009). The end of “La Muerte” usually finds the singer announcing that “when the day (of death) comes, I want there to be joy/for when they are burying me I would be very grateful if they would play me a huapango” (“cuando se me llegue el día/o quiero que haya alegría/cuando me estén sepultando/mucho agradecería/que me tocaran un huapango”) (Los Yolpakis, 2020). In their interpretation, Los Yolpakis (2020) alternatively caution to not to even chance such an encounter with death in the time of the coronavirus. “It is not time to go on vacation/Nor to party,” the trío warns. “It is time to meditate/they are going to hit us where it hurts if they end up coming around to infect” us (ya es tiempo de meditar/nos van a dar en la torre/si se llegan a infectar).

This kind of improvisational verse in the tradition of música huasteca employed during the coronavirus disease 2019 (COVID-19) pandemic provides an example of the singular and complex regional musical response to a global crisis in the Huasteca, a geo-cultural region spanning six states in central Mexico. Música huasteca is played in a trío featuring violin, a small stringed instrument called a jarana, and an acoustic bass called a quinta huapanguera. In between expressive violin passages, musicians exchange verses, frequently articulated in falsetto. These verses are drawn from a historical canon of couplets or are situationally improvised in ways remaining topical to the theme being played (the *son*)^1^. In the region, huapango simultaneously refers to the regional style of música huasteca, the events at which it is performed, and the associated style of dance. Like events, huapangos vary from private gatherings to large, semicommercial festivals. They are always deeply social events where people come together to dance, drink, eat regional cuisine, and spend time with friends and family. Such events are often organized in commemoration of a particular event in the social, religious, or political calendar, such as a birthday, a Saint's feast day, or a national holiday.

The arrival of coronavirus in Mexico catalyzed a remarkable moment of musical and organizational creativity within the Huasteca region. Newly improvised actions and compositions responding to the pandemic moment came from a diverse range of performers in the Huasteca and in its diasporic ethnosphere^2^. These musical, poetical, and organizational responses to coronavirus provide opportunities for examining a rich, an expansive, and an emergent musicultural discourse.

This article examines two interrelated aspects of the música huasteca community response to the coronavirus crisis: the growth of interactive huapango livestreams as a preexisting but newly significant space for informal community gathering and cultural participation at the onset of the coronavirus pandemic, and the composition of original verses by son huasteco performers addressing the pandemic. Both the livestreams and the newly created COVID verses reflect critical improvisatory approaches to the pandemic in música huasteca. The interactive livestreams signaled the emergence of an *ad hoc* community infrastructure facilitated by social media and a new community space fostered by Do-It-Yourself (DIY) activists. Improvised COVID-related verses presented resonant local and regional themes as a community response to a global crisis. These responses arose in sudden and unplanned ways during the COVID era and represent significant and novel uses of social technologies and virtual spaces to meet the emergent interests and needs of the música huasteca community. Digital ethnography conducted since March 2020 revealed a regional burst of musical creativity coupled with DIY intentionality, a leveling of access to virtual community spaces, and enhanced digital intimacies established across a wide cultural diaspora in Mexico and the USA. These responses were musically, poetically, and organizationally improvisational, as was the overall outpouring of son huasteco music inspired by the coronavirus outbreak.

## Methodology

This paper reflects 6 months of engaged observation of streamed and recorded videos of son huasteco performances and accompanying participant activity *via* the comments sections and chat boxes. With the understanding that the social and cultural aspects of the pandemic era are subject to perpetual change as expectations and external conditions unfold, the authors focus on media uploaded during the early stages of the pandemic, especially the early, disorienting months of March, April, and May 2020. This paper also draws on interviews conducted between June 2020 and October 2020 with Gabino “Gabo” Vera, administrator of the YouTube channel GavBroadcast, and Francisco “Chico” Gabriel Lucas, a musician in trío Los Yolpakis. All interviews were conducted remotely *via* the applications WhatsApp and Telegram, which allow for encrypted, high-resolution internet calling. While some of the insights in this paper would not have been possible without these direct, one-on-one conversations with these key individuals, the primary positionality of the researchers was as observers in the mediated digital space of the YouTube chats^3^.

Boellstorff (2013) argues that the responsibility to identify oneself as a researcher is as important in virtual ethnography as it is in the physical field. While conducting engaged observation in a livestream-adjacent chatroom, the authors adhered to a protocol of thorough self-identification. Upon entering a chatroom, the authors announced themselves by name, location, and nationality, and noted that they were writing an article about the phenomenon of online huapango events. Given the large number of participants (often more than 200) in any given livestream, and the lack of a built-in direct-message feature in YouTube's chat client, communicating with individual participants who did not publicly share their personal contact information was not feasible. Throughout the course of participant observation, the authors kept in close contact with the administrator of the channel who hosted the livestreams and their adjacent chats, asking questions and clarifying observations as needed. At present, the authors are working to expand the scope of their research of this digital community for future writing on the subject, as they are alert to the limitations of chatroom observation as an ethnographic technique. All screenshots, transcriptions of chat logs, and figures representing views, subscribers, and quantitative data were sourced either directly from YouTube while conducting virtual fieldwork or from Vera's own analytics dashboard as the administrator of GavBroadcast.

This article characterizes and offers an early theorization of an emergent musicultural phenomenon that will doubtless continue to transform in scope and character as the pandemic continues and eventually recedes into history. The authors acknowledge the possible limitations of the research conducted and written about as an initial scholarly foray into a new phenomenon during the height of the pandemic. They are invested in the continued exploration of the intersection of COVID-19 with regional and diasporic musicking and social technologies.

Over the past decade, important new research has investigated and theorized communitarian elements of the huapango huasteco and theorized translocal framings of the community oriented around música huasteca. Overall study of translocality has developed into an important subfield (Datta and Brickell, 2011). Muñoz (2013) explored how formulations of Huastecan identity as expressed in translocal festivals were impacted by state-sponsored folklore and affected by framings of race and gender in Mexico. González Paraíso (2014) examined the recontextualization of traditional repertory and performance practices in contemporary revival movements. By focusing on an emergent, diasporic community of huapangueros facilitated by social media during the coronavirus moment, this article examines newly emergent phenomena that, though linked to those examined in the above studies, ultimately extend beyond them. This study is the first examination of YouTube and Facebook as sites of social gathering and cultural content diffusion in COVID-19 era música huasteca, and the authors intend to continue to contribute to this body of research as it evolves and broadens.

## The Emergent Virtual Space for Huapango in the COVID Era

The massive musical response to the coronavirus moment was facilitated by the recent exponential growth in, and easy accessibility of, online social media sites as essential sources of musical consumption (Hepp, 2013; Nowak, 2014; Krause et al., 2021)^4^. This study elucidates the ways in which a preexisting digital space for the production and consumption of música huasteca took on new significance during a time when billions of people globally were quarantined inside their homes starting in the spring of 2020 (Heaven, 2020).

As a wide array of interdisciplinary scholarship has demonstrated, YouTube is a highly influential digital space for participatory (DIY) cultural production and music consumption. As pointed out by Yu and Schroeder (2018), “the study of the relationships between ‘global audiences' and ‘local music' deserves further investigation, particularly with regard to the interactive musical influences within the realm of internet broadcasting and distribution, where media tend to shift to nowadays, where national borders as well as ethnic identities are getting blurred evermore rapidly, and where spatial distances seem to be almost nonexistent” (2021, p. 68). YouTube's outsized significance as a *de facto* digital archive of *música del coronavirus* during the pandemic era is profound and, 1 year into the global pandemic, only beginning to be understood. The pandemic has accelerated all of these elements in ways scholars are only beginning to explore. The usage of YouTube increased exponentially during the pandemic; in some locales, usage increased by an astonishing 500% (Romero, 2020). As a platform designed to host a user-generated content, YouTube's consumers are often simultaneously its producers, with up to 500 h of original content being uploaded per minute at times (Hale, 2019). YouTube has thus functioned “as an unfiltered, bottom-up cultural archive” (Burges and Green, 2018, 137), which is particularly useful for assessing the emergent COVID musicultural ecosystem examined herein. YouTube's search capability, an essential if contested mechanism for accessing uploaded content for the platform (Geelhoed et al., 2009), can serve as an effective tool of user-driven research, offering the ability to filter content by upload date, type of video, duration, 11 different defined features, and, importantly, a robust complete keyword system.

Music is a vast content category on YouTube and an excellent case study for experiencing firsthand the complexities of transnational culture industries in the 21st century. Youtube “is now a global repository for popular music and the entry point for a vast number of listeners-consumers searching for new music” (Airoldi et al., 2016, p. 1; Cayari, 2011). Regional music genres have dominance and a wide reach on YouTube. Valcarce and Mallero (2020) note the meaningful linkages that have emerged between social media platforms and regional expressive cultures in the Hispanosphere. Research has shown that a surprisingly small amount of user-uploaded music is original on YouTube, in the range of 3–4% (Hesmondhalgh et al., 2019). Nonetheless, the surge of original compositions addressing coronavirus uploaded to YouTube demonstrates the continued centrality of user-generated musical content on the platform, even in the face of massified trends toward commercial content.

Other online social media services have a broad reach, but for various reasons are unable to recapitulate YouTube's combination of DIY ethos, ease of use, and an open sensibility that is not readily apparent to consumers and producers in other services. Hesmondhalgh et al. (2019) have examined the strictures and externalized values that are laid bare in the “platformization of cultural production” in music sites *other* than YouTube. These authors argued that “‘consumer-oriented' and ‘producer-oriented' music streaming services” like Soundcloud and Bandcamp, while professing to be putatively “independent” or “alternative” and claiming to oppose the “mainstream,” actually serve to reify central corporate sensibilities (Hesmondhalgh et al., 2019, 2; also Nieborg and Poell, 2018). YouTube shares similarities to these convergences, but these authors argue correctly that “its extraordinary multiplicity” has placed it into a separate category (Hesmondhalgh et al., 2019, p. 10). The outpouring of regional Spanish language music inspired by the coronavirus moment on YouTube is, as such, a new and consequential phenomenon. A significant amount of COVID-related music content was also posted to Facebook as live videos, as discussed below^5^.

The rise of DIY music as user-generated YouTube content means that “the boundaries between creator, producer, consumer, and audience member have blurred” (Krause et al., 2021, p. 565) in ways accelerated by a global pandemic. This acceleration of the synthetic dynamic between the global and local in music has rapidly matured during the pandemic moment. “Music, in its finest incarnations, whether in a popular song with mass appeal or in an arcane work of instrumental music cherished by a few, may have qualities hidden from others, but essential to oneself,” writes Leon Botstein, one of the first music scholars to consider the COVID musical moment. “Like sacred texts, music operates on more than one level and is a sacred possession of personhood” (Botstein, 2020, p. 356). A combination of deep uncertainty about the future, social isolation, and a shifting sense of the “normal” has facilitated an outpouring of distinctly personal musical production that reveals something intimate of the performer's inner lives, as well as the communitarian needs of the listeners^6^.

## Reticent Governments, Responsive Huapangueros: Improvisation, Digital Infrastructure, and Mutual Aid

On March 18, 2020, 25 new cases of coronavirus were officially confirmed in the entire Mexican republic, with the total confirmed cases numbering 118 (Mexico Coronavirus Map and Case Count, 2020). By this time, municipal and countrywide lockdowns were already underway throughout Europe, Asia, Oceania, and the USA (Coronavirus: The world in lockdown in maps charts, 2020). However, Mexico's political response lagged behind, with President Andrés Manuel López Obredor continuously downplaying the severity of the pandemic throughout the month of March, encouraging residents of the Federal District to “continue living life as usual” as late as March 22, 2020 (Felbab-Brown, 2020). It took another week for Mexico to implement its first nationwide restrictions, by which point the total national case count exceeded 1,000.

While the government was slow to acknowledge the need for preventative measures to stop the spread of COVID-19, artists and cultural promoters began to organize in response to the impending crisis. On the aforementioned date of March 18, 2020 DIY archivist and promoter Gabino “Gabo” Vera put out a video announcing a virtual *encuentro* (gathering) of son huasteco music. Vera's YouTube channel, GaVBroadcast, is a constantly growing repository of videos showcasing son huasteco performances. In early March 2020, the channel had roughly 190,000 subscribers. By March 2021, the subscribers had grown to 287,000. Vera, who has nearly two decades of experience working in telecommunications and network broadcasting, left his position at Televisa in January 2019 to dedicate himself fully to his YouTube channel, which generates income through ad revenue and content sponsorships. In his announcement of the virtual encuentro, Vera spoke directly to his subscribers, noting that “various huapangos have been canceled,” that “staying home is the right thing to do,” and that an online event might serve “to showcase new talents in Huastecan music” and “to help us so that our time spent at home might be more enjoyable.” This early adaptation to pandemic circumstances is a prime example of the improvisational flexibility of the online huapango community and its digital infrastructure. At a moment when governments still struggled to effectively respond to the coronavirus outbreak, community content creators such as Vera put forth solutions to a problem while also establishing a new normative connection between social responsibility and artistic engagement.

As a participatory DIY archive, GaVBroadcast's *ad hoc* content strategy actively mirrors the improvisationality of the trovadores who it documents and promotes. For years, Vera managed his channel as a hobby while working in Mexico City, recording performances in private homes on occasional return visits to his natal Huasteca. The channel was created in 2007, long before YouTube attained the prominence and ubiquity it holds today, and subsequently grew with the platform. Vera was a relatively early adopter of the livestream format as a means of broadcasting local performances to a global audience of huapango enthusiasts. The YouTube livestream platform includes a chat feature that allows for viewers to engage with the broadcaster and the performers, encouraging a dynamic informality that mimics the social ethos of in-person community performances. These casual and personalized performances cater to a diaspora that extends far beyond the geographical Huasteca. However, as the pandemic took hold and people across the globe entered into self-isolation, this digital participatory infrastructure came to serve an increasingly expansive audience of huapango enthusiasts.

Multitudinous examples of mutual aid networks emerging during times of ineffectual governance can be found throughout history (Benatar and Brock, 2011; Hilhorst, 2013; Orduña-Malea et al., 2020). Moments of crisis often serve as catalysts for communitarian initiatives that otherwise may be deemed unnecessary, untenable, or unwelcome (Spade, 2020). In her recent trade book on community resilience in the face of disaster, Rebecca Solnit notes that collective responses to crisis can provide “a glimpse of who else we ourselves may be and what else our society could become” (quoted in Garner, 2009). The following section describes how a vibrant cultural ecosystem of performers, consumers, and digital content creators emerged to meet the needs of a community in crisis.

## The Diy Huasteco Livestream as Communitarian Space

Early in the pandemic, numerous tríos huastecos performed livestreamed concerts on Facebook. Trío Santuario Huasteco started their April 24, 2020 stream with a simple statement of “quédate en casa con música huasteca” (stay at home with Huasteco music) and other exhortations to “stay home with huapanguitos …it's worth asking.” Listeners discussed their hometowns and thanked public officials with comments like “greetings to ‘Toro Requesón,' who from his trench, is doing an extraordinary job with the Ministry of Health and all the staff under his leadership” (https://www.facebook.com/triosantuario.huasteco.7/videos/855159068336523/).

The events, which generally lasted 90 min but often went on for hours, were variously coded with hashtags such as “#huapangos,” “#QuedateEnCasa,” or, more generically, “#EnVivo.” The first two labels spoke to the pandemic moment for the Huasteco diaspora most directly, and rapidly became the descriptors of choice. Despite the different origins and timings of the livestreams from the start of the pandemic, standardization in coronavirus-era online huapangos emerged quickly and noticeably. Musicians grew to accept their evolving role of supporting community cohesion and providing entertainment in the midst of restrictions, thereby establishing a new standard of performance and audience engagement. By May 8, 2020, longer encuentros with multiple groups had become increasingly common. Groups Eco Potosino, Tordo Huasteco, Desafío Huasteco, and Santurio Huasteco shared a “Huapangos Huasteco” event, with each trío performing from their respective communities, their performances digitally stitched together (https://www.facebook.com/permalink.php?story_fbid=1177044319301548&id=100009879738811).

Among the groups who engaged with this novel format, new norms of performance emerged relatively organically, though not all musicians were quick to acclimate. In general, tríos came to adopt a relatively consistent presentational style and adhered to a widely shared mode of audience interaction, which was distinctive from other pandemic era livestream performances of traditional music in styles as varied as conjunto Tejano, old time Appalachian, and Western Swing, as observed and participated in by the authors. In the COVID virtual huapangos, the musicians in many ways positioned their music making as a service to a community in crisis, explicitly framing their performances as expressions of resilience in the shadow of the coronavirus.

Two months into the pandemic, Trío Sentimiento Huasteco played a live set on Facebook for more than an hour, standing on a small stage. They listed their representative's phone number for potential future in-person gigs, but mostly responded to listener requests. This was a pioneering livestream version of the COVID era virtual huapangos already happening on YouTube, hosted by channels such as GaVBroadcast, QuerrequeFilms, and others. As is common at in-person huapangos, at the virtual events listeners asked for favorite sones; Antonio Emperador asked for “un huapango favor La Azucena bella.” Listeners checked in from Coyutla, Veracruz; Riverside, California; Tamazunchale, San Luis Potosí; Mexico City; Huejutla, Hidalgo; Matamoros, Tamaulipas, and numerous other places. In between the sones, a mask-wearing narrator came out and read the comments from the live chat off his phone to the trio, along with greetings and requests. All of the comments from the chat were read, signaling the egalitarian ethos of the gathering. Not all trios were immediately comfortable with this new performance model. Trío Sentimiento stood stone faced and barely responded to the list, although the quinta player did smile (https://www.facebook.com/watch/live/?v=2676354835945249&ref=watch_permalink).

Tríos huastecos delivered performances in dynamic and constant interaction with viewers *via* their smartphones. In the digital COVID huapangos, it was not uncommon to see all three members of a trio looking at their phones during the livestream at the start, and at nearly every moment not spent playing an instrument. An example was trío Fuerza Imperial's livestream where the members of this huapango arribeño^7^ group started the stream but did not start playing music for a static 5 min while a phone conversation and children playing could be heard in the background. Five minutes of silence does not seem like a long time unless one is sitting watching a motionless screen. Performers traditionally (or not during a pandemic era streaming event) would recognize this long gap as “dead air,” whereas in the context of the pandemic era stream it felt as an essential connection with the audience. It was, in the language of those studying music cognition, more of a pause—an opportunity for the audience to “be with” the trio in a moment adjacent to but not part of a staged performance. Indeed, with 2.3 thousand views, the video was widely watched; the pause did not drive away the audience. Live streamed videos are archived as originally streamed. The pauses remain built into the performance, integral to the space of the pandemic performance, which lives on in the internet as a node of community interaction (https://www.facebook.com/trio.acuarela.5/videos/672973366920749/).

In between *sones* during pandemic livestreams, each trío interacted directly with audience members by reading names, recognizing regions, and states of origin (generally in Mexico) and of current residence (often in the USA). Each group responded directly to requests for songs. These requests were listed in the chats during the stream, usually accompanying a compliment, a shout of encouragement, and a mention of the origin of the requester. The tríos then would read some, or all, of the comments to the listeners, serving to reinforce the connection between the audience and performer. Trío Amanecer Huasteco's June 13, 2020 livestreamed followed the established practice. This was a noticeably professional group, with wireless ear microphones and heavily amplified sound. They had listeners from Houston and Paris, Texas; Matamoros, Tamaulipas; Tequisquiapan and Ahuacatlán De Guadalupe, Querétaro; Puerto Rico; Georgia, Cuetzalan, Puebla, and other places. Listeners were recognized, their comments read and discussed, and their requests fulfilled (https://www.facebook.com/watch/live/?v=636191986967495&ref=watch_permalink).

While the regional repertory is quite extensive, there was in fact little variety observed among the set sones performed at most live-streamed huapango events. The most commonly performed sones (El Querreque, El Gusto, La Leva, El Cielito Lindo) were widely known and beloved within the repertory. All feature a standard stanza format that allows improvisation. Tríos also played a wide variety of requests as they came in, so it is not uncommon to hear other sones depending on the group and the origin of the listenership. The responsiveness of the groups to the viewers constituted a part of the improvisational core of the livestream space as it did in an in-person huapango. As is typical in live, in-person events before the pandemic, there were no set lists; the performances changed and could go in any direction within the boundaries of the style's conventions at any point in the night. This responsiveness and changeability were the key features of the live streams, and a draw for both viewers and performers.

Trío Cantores del Alba placed a live unamplified set in a living room on May 31, 2020. Like all the other groups, they spoke with fans appearing in the chat, answered a large number of song requests, and were extremely interactive with the listeners. Younger than many groups, Cantores del Alba were clearly relaxed in the virtual environment and deeply engaged with the large crowd of 2.6 thousand viewers. This contrasts with some of the older groups whose discomfort in the digital space was evident in early livestreams (https://www.facebook.com/watch/live/?v=2987014701385647&ref=search). They were highly aware of the differences of this kind of performance in the new COVID era, posting a video on YouTube, which presented dancing to the song “China del Alma” at the “Feria De Las Flores 2020” festival in Huauchinango, Puebla the prior March “antes del #covid” (emphasis original, Cantores del Alba, 2020).

The COVID huapango live stream of trío Perlitas Queretanas, a very accomplished all female group, moved at steady and even relentless place. The trío, representing the gender transformation currently occurring in son huasteco with the rise of all female groups as explored in earlier contexts by Muñoz (2013), followed the new, but now solidly established mode for virtual huapango events during the COVID era. Their video started without any introduction, with the camera centered on a couch where the jarana player scrolled on her phone as the violinist tuned. Although only allowing ~1 min between songs, the trío dutifully recognized their listeners and their requests. The jarana player is seen texting on her phone between each son, responding to viewers in real time. This stream was presented under the auspices of a Texas-based organization called fittingly, Huapango Sin Fronteras (“Huapangos without borders”). This organization puts on an annual festival of son huasteco and huapango arribeño in Austin, Texas each May. In 2020, the festival was held virtually with a series of performances livestreamed onto YouTube from stages in Texas and Mexico (Huapango Sin Fronteras Virtual, 2020).

Trío Perlitas Queretanas kept a steady, but focused, chat in between songs. Their breaks were kept to barely a minute, during which times the musicians called out the names of listeners in long lists, along with origins of the people in the chat. Viewers and chat participants logged in from large cities such as Monterrey and Guanajuato, communities in the Huasteca region such as Aquismón and Xilitla in San Luis Potosí and locations in the USA including Dallas and Houston in Texas, Charlotte and Durham in North Carolina, and Salinas, Gilroy, Madera, Oakland, and Oxnard in California (https://www.facebook.com/watch/live/?v=394495201669636&ref=search).

By cultivating a communitarian sensibility within the space of the virtual huapango, these events came to constitute complex assemblages of social meaning and connection. The huapango streams created a spatialized normative frame for community connection and sustenance during the quarantine. The featured tríos promoted these events themselves, in addition to a community-based groups like Huapangos Sin Fronteras and California-based FJ-Xichu Promotions, which put on a “Huapango Facebook Live” on November 26, 2020, with four groups, three of them are all female tríos huastecos (Perlitas Queretanas, Nueva Herencia, Palomitas Serranas), and a duo called Toño Jimenez. These groups represent the outgrowth of a now established, concerted initiative by huapanguero musician educators to teach and encourage young women to perform as musicians. Although female musicians now play such a central role as to make the presence of all-woman groups unremarkable, their visibility nonetheless challenges patriarchal narratives that point to the old man as the archetypal son huasteco musician (Muñoz, 2013).

Virtual huapango events during the COVID are distinctively informal and challenge assumptions about the necessity for extensive, diversified branding when crafting a cultural product that keeps participants engaged. The formalized, market-based approach to pandemic music holds that successful events require advance planning, novelty, and difference. For example, the CEO of a commercial streaming concert service called LoopedLive declared “‘if you do the same thing over and over again, people won't want to tune in”' (quoted in Blake, 2020). Yet, for digital COVID huapango events during the pandemic, it was exactly this combination of malleability and consistency that produced and satisfied audiences. Quarantined listeners who logged into virtual huapangos and participated in their chats were in search not of novelty, but of the familiar.

The dominant style of the virtual COVID huapangos was almost studiously non-theatrical. This stood in sharp contrast to the virtual events and the festivals and what González Paraíso calls “cultural projects” staged in the Huasteca, which served as a “medium for community building, cultural transmission, and intergenerational communication” and exchange of salient “codes and ideological symbols” (González Paraíso, 2014, p. 5, 150). At these events, standing tríos wearing traditional costumes performed with studied gravitas and enacted Huasteca identity in pronounced ways. In contrast, the virtual COVID huapangos featured performing tríos sitting on couches, standing loosely outside or inside garages, or in makeshift stage areas carved out of living rooms, with bay window curtains serving as the stage and lamps as lighting. Not uncommonly, given the studiously unprofessional approach to the streams, sound was often decidedly unprofessional, with some instruments too loud, though streaming quality varied across channels and generally improved over time. Groups often played their sets dressed not in the matching guayaberas or vaquero suits sported by tríos in conventional festival settings, but in T-shirts and jeans. This casualness was in keeping with a broader style of live streamed performance of vernacular musics, where the informality of the early pandemic era—with people thrust into at-home isolation with uncertainty and loneliness—was enacted in the videos, referenced consistently, and widely accepted. Making, watching, and capturing these types of videos produced social capital that could be shared among the communities of enthusiasts assembled (Colburn, 2015). The feelings produced for the viewers were of having arrived at a friend's house for an intimate get together or for a backyard pachanga. “#QuedateEnCasa” meant stay at home, but also come to a new space of the trío during the pandemic, the home of the huasteco diaspora.

By allowing for a real-time interchange between performers and viewers in diverse locales, livestreamed huapangos during the COVID era addressed the articulated needs of a coherent audience that continued to grow in intimacy as these events became increasingly regularized. Gupta has written about the deep, human pull toward fellowship in the arts and music during a time of crisis. “The antidote of musical solidarity in a time of coronavirus provides a joyful reminder of the deep human will to always find our way back to one another” (Gupta, 2020, p. 596–597). These streams differed from the nightly huapangos put on by GaVBroadcast and other channels because of the spontaneity of the presentation and the directly interactive modality. Nonetheless, they tracked a similar trajectory in terms of the community created in the streams and the service provided to the audience of members in the Huasteca diaspora. González Paraíso (2014) has explained how interaction with the audience is a central objective of live son huasteco performances. Livestream son husateco events of the COVID era recapitulated the core “cultural politics of representations of place, space, and landscape” (Rose, 2016, p. 336) found in such traditional, in-person events. During the coronavirus pandemic, the connections created in virtual spaces were essential recreations of the long-standing participatory culture fundamentals of huapangos.

Coronavirus huapango streamed videos present a true sense of intimacy as a core component of the #QuedateEnCasa videos. The videos are livestreamed *via* the internet, but this streaming also provides a broadly immersive experience for the audience, pushing viewers beyond the status of passive consumers and inviting them to participate. Scholars writing about livestreaming have recognized this immersive flow. Gupta writes “In this time of coronavirus, examples of therapeutic music-making unceasingly flow” (Gupta, 2020, p. 596). Rautiainen-Keskustalo and Raudaskoski (2019) explained that in the “spatial formation of the terrain” of the livestream, “musical material, which moved over the spaces, [and] the institutions opened up to ‘flowing' and ‘moving' when live-streaming established a connection between them.” The livestreamed space could be understood then as a “mediated community” built upon a mobility paradigm (Rautiainen-Keskustalo and Raudaskoski, 2019, p. 469–483).

Coronavirus music livestream assemblages produce and perpetuate a space for intimate community building. The scholarly literature on intimacy in online social media spaces is largely connected to personal sharing of private and often sexual details (Waugh, 2017; Dobson et al., 2018). Much of the most important literature related to intimacy in online social media platforms comes out of “queer and feminist theory of intimate publics” (Dobson et al., 2018, p. xx). Here, we consider the role of online intimacy as defined as ‘“the affective encounters with others that often matter most”' (McGlotten quoted in Dobson et al., 2018, p. 4) in musically intimate communities of diapasonic affinity during the pandemic.

When the stream is viewed on the large screen of a smart TV or on a desktop with a large screen, the facial expressions of the players are magnified and intensified. The viewer notices smiles, raised eyebrows, glances, and other looks at the screen which fee for all intents as glances directly at the viewer. The focal point of the players' gaze is of course the lens of the streaming camera, exactly where the eyes of the viewer are. There is an illusion of intimacy and reality of intimacy, which is not captured in other formats. Musicians are arrayed on a couch, as if playing on the other side of the living room. The informality with which the livestreamed huapangos were presented and performatively delivered is therefore highlighted and is definitely a large component of the draw of the whole experience. As Lambert has argued in dissecting social capital in Facebook before the pandemic, in creating intimacies online, argues “the performative element of social capital is central” (Lambert, 2016, p. 2,560).

This sensibility is magnified and seen most obviously when the listener is subsumed into the livestream and the chat, which is a key component of all streams and of the digital spaces created to meet the community needs during the pandemic. Lambert argues “in making reference to various musicians which take on specific meanings for this group, these friends simultaneously perform their collective stock of social and cultural capital. Note the light and playful tone. Social capital imbricates through this gregarious form of public intimacy” (Lambert, 2016, p. 2,569). The connections created online in these digital spaces of the pandemic clearly presented people seeking friendship and intimacy, which was especially clear in the ways these participants jointly imagine, long for, describe, and anticipate the post-pandemic return to in-person events like huapangos, the *d*í*a de plaza* (described below), and general family gatherings. Online relationships can be far more profound, meaningful and sustainable than the uninitiated might think, as Lai and Fung (2020) found in their recent study of the social ties formed in virtual spaces.

While some have lamented the “the deafening silence of the remote audience” (Geelhoed et al., 2009, p. 5,583), this fate is entirely disarticulated in the digital huapango space, in which both viewers and performers are participants with real agency. Participant observation in the livestream chats demonstrates that people are quite the opposite of silent. If anything, the chat is constantly active and considered an essential component of the stream. This utility during the digital COVID huapangos tracks along with what occurs at in-person huapango events. There are constant recognitions of individuals, mentions of birthdays and anniversaries, appeals to place names and regional identity, and shouts of “¡Puro Huasteco!” and “!‘ajuua!”

## Te Pido Atención Porque Soy Trovador: Situational Improvisation in Son Huasteco

All of the coronavirus music is, in its own way, original. All of it idiosyncratically grapples with the terrors, challenges, and unknowns of the COVID moment. These musical approaches are particularly clear in the verses responses to the crisis among *trovadores* huastecos, the song poets of central Mexico. Through a robust network of DIY archivists and online content creators principally on YouTube, the tradition of improvisational verse commentary in the Mexican Huasteca region came to be directed at the COVID-19 crisis in the spring of 2020.

Perhaps, the most defining quality of verse in Mexican sones is liberal invocation of situational improvisation. Sturman (2015, p. 105–106) comments “the poetic verse of the son may feature classic lines of text, but singers often improvise verse while performing.” Béhague notes that “in the regional sones of Mexico, improvised *coplas* are frequently the necessary adjunct of a successful performance.” Logically, the most impactful responses to the COVID crisis include improvisation. Béhague highlights how, in the “Mediterranean [derived] song-duel tradition in Latin America,” performers employ “textual improvisation” to “tease [one another] or quarrel verbally” (Béhague, 1980, p. 119). Because of this emphasis on spontaneity in verses, “standard melodic and rhythmic formulas are used to minimize the need for… musical extemporization.” Sánchez Garcia (2009, p. xix) affirms that “the improvisation…frequently executed by vocalists…is a distinct element of the Mexican son, although it varies in frequency and intensity across regions.” The improvisation even about novel events occurs within a bounded framework.

The narrative poetics of many Mexican vernacular musics situate the lyricists as both positivist purveyor of information and normative emblem of identity. This tendency has been most thoroughly explored in studies of the corrido, a type of narrative ballad from the Mexico-US borderlands. Chamberlain (2003) presents corrido singers as “describing singular events” while also serving as “the voice of a community.” The distinct positionality of the “embedded storyteller” who employs poetics to simultaneously share news and affirm identity extends beyond the corrido and can be observed throughout the vernacular and traditional musics of Mexico, including in son huasteco.

If skilled lyricists (*trovadores*) are present at a huapango, they will be expected at some point in the evening to take the stage and improvise verses in reference to the event's *raison d'etre*. These verses can range in tone from praiseful (e.g., at a huapango commemorating the Ascensión of the Virgin Mary, the *trovadores* bestow honorifics and compose prayers in *copla*-form) to deprecating (e.g., at a huapango celebrating a man's 50th birthday party, his friends tease him for supposedly being “over the hill,” etc.). When they have earned the attention of the crowd, *trovadores* may also take license to use their momentary visibility as a pulpit for sharing personal news, praising the hosts, asking a question or a favor, making a pass at a romantic prospect, opining on the state of the wider world, shouting out their hometown and family, or responding to other *trovadores*.

The *trovador* is both a harbinger of news and an embodiment of regional identity, mimetically linking novel information to recognizable forms of vernacular music and poetics through improvisation. Indeed, improvisation is so integral to son traditions that it has its own lexicon among son practitioners of the situational improvisation of verses and the poetic material that arises from the practice. The poet singer who is skilled at improvisation is a *trovador* or *versador; coplas, quintillas*, and *decimas* denote rhyme schemes common in improvisations, and sexually charged (*albur*) or sociopolitically dissident (*oblicua*) double meanings have their own dedicated terms. Throughout the first global wave of the pandemic, trovadores produced volumes of improvised COVID-19 verses that applied established themes to a new phenomenon.

For their examination of verse responses to the coronavirus in digitally circulated son huasteco, the authors of this paper have selected 10 examples that showcase a variety of the techniques and approaches covered throughout this emergent body of work. All 10 examples, which are listed in Table 1, are housed on YouTube and were uploaded in April of 2020. These selections were chosen not only for being representative of the general response but also for showcasing particular distinguishing factors that make them noteworthy.

**Table 1:** Selected examples of sones featuring COVID-19 verses that are discussed in this article.

**Name of son**	**Name of trío**	**Location**	**Date released**	**Link**	**Distinguishing factors**
La Muerte (Death)	Los Yolpakis	Ixcatepec, Veracruz	April 14, 2020	https://www.youtube.com/watch?v=7oSdF7UotQg	Verses sung in Spanish and Nahuatl
El Gustito (the little pleasure)	(Augusto “Atte” San Agustin, 2020)	Playa del Carmen, Q.R. (originally from Huejutla, Hidalgo)	April 6, 2020	https://www.youtube.com/watch?v=TgPXDAVZCWE	Singular musician multi-tracking all three instrument parts from home
El Querreque (the woodpecker)	(Trio Andante Huasteco, 2020)	Ciudad Valles, San Luis Potosí (SLP)	April 6, 2020	https://www.youtube.com/watch?v=LDHPxqdN79E	Performed in a public square
El Querreque	Trío Juvenil Las Orquideas de Valles	Ciudad Valles, SLP	April 3, 2020	https://www.youtube.com/watch?v=S2kQm_8yXlA	Juvenile, all-female trío
El Querreque	Trío Cenzontle de Tamalin	Tamalin, SLP	April 2, 2020	https://www.youtube.com/watch?v=8IPbBA8EMmE	Video part of a local news broadcast to warn community of COVID
La Leva/The Conscription	Various trovadores (channel: Cotorro Huasteco)	Various	April 22, 2020	https://www.youtube.com/watch?v=jxeKWiQJ5Eg	Collaboration between various trovadores in disparate locations
El Perdiguero / The Retriever (dog)	Trío Zarpazo Huasteco	Zozocolco de Hidalgo, Veracruz	April 7, 2020	https://www.youtube.com/watch?v=24gOM3uMvXo	Filmed in 4K video
El Gustito	(Trio Encuentro Huasteco., 2020)	Queretaro City, Queretaro	April 8, 2020	https://www.youtube.com/watch?v=ZBdKmllApcA	Urban trío performing on electric instruments
El Huapango del Coronavirus	(Los Venaditos, 2020)	Citlaltepetl, Veracruz	April 7, 2020	https://www.youtube.com/watch?v=hfdV-rkEO-Y	Original musical material; two musicians rather than 3
El Querreque	(Trio Esencia Huasteca., 2020)	Tantoyuca, Veracruz	April 1, 2020	https://www.youtube.com/watch?v=L5eXvyZndb4	Earliest of selected videos

This selection showcases a wide spectrum not only of response types, but also of groupings, performance styles, and identities present within the son huasteco community. Here, one can encounter performers singing in Nahuatl, groups representing rural, urban, and diasporic positionalities, male and female musicians, a wide range of ages from children to elders, outfits ranging from casual to flashy, and heterogenous levels of video resolution falling anywhere between 240 and 1,080 p. The verse responses found in these 10 examples, while original, tend to draw from similar thematic vocabularies. The following section will examine how four of these themes: (A) news sharing, and calls to collective responsibility, (B) localisms and the invocation of objects, (C) comedy and wordplay, and (D) dialog with the listener—extend the tradition of trova to the emergent crisis of COVID-19.

## Theme A: News Sharing and Calls to Responsibility

As previously noted, various forms of Mexican son have historically been instrumentalized for the purposes of disseminating important information (Heath, 2015). Son huasteco serves such a function as well, albeit often on a more localized scale. When improvising at a huapango, the trovador is granted license to make announcements that are considered worthy of sharing to a wider community. As one example, one of the authors of this paper attended an in-person huapango in February 2019 at which trovadores announced such varied happenings as a new pregnancy in the host's extended family, an accident on a major road in the region, and a gaffe made by then US President Donald Trump.

At the outset of the COVID-19 pandemic, *trovadores* were quick to instrumentalize the huapango as a format for transmitting both positive and normative information on the virus. About 4 of the 10 examples selected herald the virus' origins in China, and two make note of how challenging the illness can be to treat. Trío Juvenil Las Orquideas highlights the severity of the virus, warning that “aspirin does not cure it” (no lo cura la aspirina) and that “it could cause death” (la muerte pueda causar). Similarly, Los Venaditos note that “they tell us that (the virus) is lethal” (nos dicen que es muy letal) and that “you will end up very badly” if you don't believe in it (si tu no lo crees, vas a terminar muy mal). At a first glance, these pronouncements may not seem radical, but they stand in contrast to the positions of various leaders throughout Latin America, such as Brazilian President Jair Bolsonaro's claim that the coronavirus is “little more than a cold” and Mexican president Antonio Manuel Lopez Obrador's insistence on attending in-person events without a mask through late March (Phillips and David, 2020). In Trio Zarpazo's COVID-inspired rendition of El Perdiguero, the vocalist acknowledges the doubts that many Mexicans, including himself, had felt regarding the necessity of quarantining, but how he ultimately came around to understanding its severity.

“Some say it's not so/And they mistakenly comment (pues unos dicen que no/y por error comentaron)

I used to think similarly/but the news informed us (lo mismo pensaba yo/pero las noticias informaron)

That from the analyses arose/Various confirmed cases” (de los análisis salieron/varios casos confirmados) (Trio Zarpazo Huasteco, 2020).

In several examples, didactic verses with calls to “stay home” (quédate en casa), “wash hands” (lávense las manos), and to “not go out without a face mask” (no salgas sin cubrebocas) are performed in contexts that corroborate the performer's role as herald of news and authority on right action. In Trío Andante Huasteco's video, the trío is seen performing in an iconic plaza in Ciudad Valles, the most populous city in the Huasteca. As people mill around in the background, the trio's jarana player and primary vocalist announces that “attention is sought from the whole little nation” (la atención se solicita a todita la nación) and that “precautions are required” (precauciones se necesitan) including “to wash your little hands with water and soap” (que se laven las manitas con aguita y con jabón). The use of diminutives (“todita la nación”; “que se laven sus manitas”), a common way of bestowing affection or signaling cuteness in Mexican vernacular Spanish, lends the performance a lightness of mood while affording the vocalist an opportunity to share a message that is deemed necessary. Trío Cenzontle de Tamalin's video, which is also highly didactic in its lyrics, is shown as part of a local news program. At the beginning of the clip, before the trio begins singing verses urging the listener to “stay in your house” (quédate en casa), an anchor announces the trio accordingly: “the theme of COVID-19, also known as coronavirus, has made its rounds across the globe. In the municipality of Tamalín in Veracruz, Trío Cenzontle Huasteco, comprising Eddie, Julián, and Victor, to the rhythm of El Querreque, sing about the coronavirus and make recommendations to the populace to observe sanitary measures” (el tema del Covid-19, o también conocido como coronavirus, ha dado la vuelta al mundo. En el municipio de Tamalin en Veracruz, el Trío Cenzontle Huasteco; integrado por Eddie, Julián y Víctor, al ritmo del Querreque, les cantan al coronavirus y hacen recomendaciones a la ciudadanía de respetar las medidas de sanidad) (Trio Cenzontle de Tamalin, 2020).

It cannot be understated that this style of peer-to-peer encouragement to observe preventative hygiene arose before governments in Mexico began to announce formal restrictions. By linking the urgent need for social distancing and “sanitary measures” to popular local patrimony, groups such as Trío Andante Huasteco and Trío Cenzontle de Tamalin are important actors in the formation of new norms, which link public health to community well-being. The following section will describe how many trios furthered this norm-setting situate the global pandemic in terms of local significance.

## Theme B: Localisms and Regional Contextualization

As an unprecedented worldwide crisis, the scope and scale of the coronavirus pandemic are, simply put, incomprehensible to any single individual. As such, while the pandemic's impact is global, distinctive regional responses have arisen throughout the world. These localized frames for understanding the pandemic are rooted in the essential aspects of life most disrupted by the virus in a given region. This is certainly the case for the son huasteco response, which invokes social, economic, and linguistic localisms to render the COVID moment legible to a population that conceives itself as far from the metropoles from which the virus initially spread.

For example, three of the selections make reference to *d*í*a de plaza* (plaza day). The *plaza* is a temporary market that occurs on regularized days (typically Sundays and sometimes Wednesdays) in town centers throughout the Huasteca. Since many municipalities in the Huasteca consist of dense, colonial *ciudades* surrounded by a hinterland of rural *comunidades* and *ejidos*, the *d*í*a de plaza* allows for families from the periphery to sell and shop for foodstuffs, artisan wares, and specialized services (tailoring, carpentry, etc.) in a centralized location. The *plaza* is an essential part of life in most municipalities throughout the Huasteca, especially those with populations too small to support a daily market or grocery store. It provides economic sustenance for rural farmers and artisans, goods and services to residents of the town, and a setting for casual socialization. However, like other stages for large gatherings, *d*í*a de plaza* is a potential site of mass viral spread.

The violinist of Trío Cenzontle de Tamalin acknowledges in his opening verse how “in my town there is no more plaza, and it affects my good people” (en mi pueblo no hay plaza, y afecta a mi gente buena), speaking to how central the *plaza* is to the socioeconomic fabric in his community. He continues by “asking all the community” to “stay home” even though “it grieves [him] to say it” (aunque mi verso aquí me apena… se lo pido a toda la raza/que en esta cuarentena, pues quedate en tu casa) (Trio Cenzontle de Tamalin, 2020). By acknowledging the difficult sacrifices that observing quarantine entails, this *trovador* affords himself legitimacy by way of empathizing and placing himself level with his audience. Likewise, the huapanguera player in Trío Andante Huasteco encourages its listeners to “not go to the plaza day” (no paseas en el día de plaza) implying that the weekly occurrence has not been canceled in all communities. In this exhortation, the *trovador* is encouraging his listeners to take personal responsibility for their actions. Moving beyond the mourning of closures, this message articulates the need for individuals to exercise their own agency when public policy is irresponsibly lax.

The nearly 10-min long rendition of La Leva “with COVID-19 verses” (con versos covid-19) consists of vocalists responding to one another through clips recorded on home webcams, with footage of COVID-related news reels spliced between verses. This extended improvisation session features many distinct localisms. At one point, one of the *trovadores* speaks of changes in commodity prices that occurred congruently to (though not necessarily because of) the pandemic: “Well, the price of gasoline fell/But there is not a happy ending/The tortillas got more expensive/We're going to run out of corn” (pues, bajó la gasolina/pero no hay final feliz/ya subieron las tortillas/se va a acabar el maíz) (Cotorro Huasteco, 2020).

The choice of the two commodities mentioned is telling. Both of these goods serve an essential role in the local social economy, with corn a staple of the local diet and gasoline serving an essential role in the region's informal transportation system. Whether or not these price shifts were directly related to the pandemic, linking an incomprehensibly vast pandemic to a tangible change in the regional economy helps to demystify the connections between the global and local.

**Figure 1:**
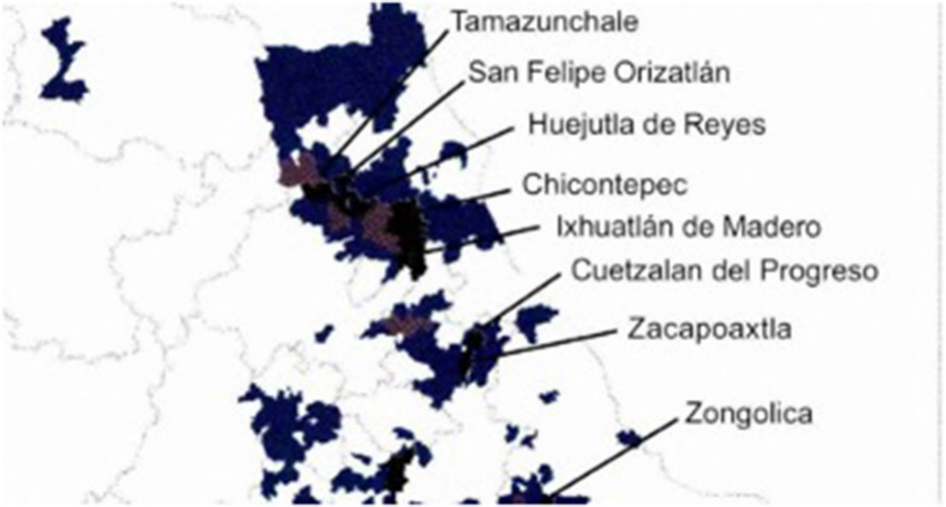
Municipalities with more than 1,000 Nahuatl speakers (shaded) and cities with more than 5,000 speakers (labeled) in the Huasteca (INEGI, 2014).

Many examples make use of localized vocabulary and argot that very clearly situate the performance in a Central Mexican cultural space while simultaneously referring to the globalized spaces of the pandemic. The most explicit example of this phenomenon can be found in Los Yolpakis' rendition of La Muerte, in which they sing verses about the coronavirus in Spanish and Nahuatl, discussed in the opening of the article. Singing verses in Nahuatl serves several purposes: it provides legitimacy to a group professing to represent a locality with deep indigenous identity, allows for the transmission of messages to individuals whose primary language is Nahuatl, and demonstrates the flexibility of heritage languages for developing ways to discuss novel events. Figure 1 presents the areas where Nahuatl is widely spoken.

Even when performing in Spanish, *trovadores* integrate vernacular language that is often rooted in indigenous calques. Consider the following from La Leva: “With this strange virus/A great (fuss has been made/the people are like a herd/and the toilet paper leaves us on edge/still, wanting to go to the bathroom/but only “corn cobs remain” (con este virus extraño/se hizo un buen borlote/la gente como rebaño/y el papel lo deja aflote/con ganas de ir al baño/y quedaron puros olotes) (Cotorro Huasteco, 2020).

As noted by the *Gran Diccionario de la Lengua Española*, the two instances of vernacular Spanish above (borlote as fuss and olote as corncob) are distinct Mexicanisms that are not widely used or recognized throughout the rest of Latin America. In fact, the word *olote* again highlights the cultural centrality of corn, and can be directly traced to its antecedent, the Nahuatl word *olotl*, which carries the same meaning. In another clever linkage between the global and the local, this *trovador* employs a localism (the word for the corn cob after the husk and kernels have been removed) to make a joke about a toilet paper shortage that was experienced at the international level. Alas, New Yorkers, Parisians, and Tokyoites may all have been left with mere empty toilet paper rolls, while Huastecos were left with *olotes*.

## Theme C: Humor, Double Meanings, and Comic Relief

As shown with the example of *olotes*, many of these examples are full of humor and wordplay. Comic relief has been used as a literary tool to counterbalance tragic narrative arcs since at least Shakespeare's time (Nason, 1906) and as a coping mechanism during recent times of crisis such as the aftermath of 9/11 and the AIDS epidemic (Christiansen and Hanson, 1996; Achter, 2008). The social acceptability of applying comedy to tragic situations can drastically vary across time and culture, but it is evident from the outpouring of humorous verse in *huapangos de coronavirus* that this practice is far from taboo in the Huasteca. Indeed, Mexico has a deep tradition of vernacular humor through double meanings. Sexually suggestive entendre, known as *albur*, is ubiquitous in Mexican popular literary forms. Political double meanings, known as *l*í*rica oblicua*, are also employed to veil statements that ridicule governments and powerful interests during instances where critical speech might provoke retaliation.

Several of the selected examples include instances of comedic entendre. Los Yolpakis note that the virus could bring “la corona de la muerte santa” [the crown of holy death, or the *corona(virus)* of holy death). In “La Leva,” one *trovador* states that the pandemic situation “ya hizo subir los huevos” (which may mean “has already caused eggs to increase in price,” but also could mean “just made my testicles retract”), coding a lewd joke within a bit of mundane commentary. Jokes about being “trapped inside” with one's spouse, failing to adequately clean the house, and gaining weight during quarantine are also found throughout the selected videos. In “La Leva,” one *trovador* warns his listeners that he is growing fat from excessive drinking in quarantine (“y yo me estoy engordando/ pues yo por tanto tragar”).

Humor is also established through the absurdity of contrasts. In “La Leva,” one *trovador* acknowledges that “the musicians here are all talented, though also all unemployed” (grandes músicos aquí, aunque todos desempleados) and that “we are all anxious about this strong virus, and to top it off, the President canceled the gigs” (el virus anda bien fuerte/a todos los preocupo/y de paso el presidente/las tocadas canceló) (Cotorro Huasteco, 2020). These statements, while ostensibly positing the painful reality of economic struggles during a global crisis, are uttered in jest, demonstrating how comic relief can be used as a coping technique in a time of multifaceted crisis.

## Theme D: Dialog With the Listener and Calls to Collective Unity

A fourth quality of the son huasteco response to coronavirus that requires attention is the dialogic nature of the performance style. In several examples, *trovadores* use terms such as “my friends” (mis amigos), “my brothers,” (mis hermanos), and “my good people” (mi buena gente) when addressing the audience, establishing a relationship of conviviality and care between the performer and listener. Verses often alternate between a formal, proclamatory tone and an intimate voice that speaks directly to whoever is listening. Los Yolpakis break down this third wall even further by singing “entiendan entonces, cabrones, es tiempo para meditar” (“understand then, bastards, that it's time to think”). In Mexico, the term *cabrón* (literally “big goat,” or bastard), is technically vulgar and can be offensive in many contexts, but is also used endearingly among confidants to cajole or single out one another. By employing such a term in a public-facing video, Los Yolpakis signal that they consider the listener to be worthy of this intimate code-switch.

Calls to collectivity are also extremely prevalent in improvisations about coronavirus. In 6 of the 10 selected examples, *trovadores* invoke some form of collectivity. Trio Andante Huasteco proclaims that “we must take this step by step, and like a good Mexican… follow the guidelines,” (hay que irnos paso a paso/y como un buen mexicano/a las medidas hazle caso) tying the observance of sanitary measures to a sense of national pride and shared values. In “El Gustito,” Augusto San Agustín asks his listeners to “not be negligent” (que no haya negligencia) and reminds them that “we will fight this evil” and “if not, it will reach us all” (combatamos este mal/si no a todos nos alcanza). Trío Esencia Huasteca announces that “we must unite for the cause” (a la causa nos sumamos) and that individuals in the community “must take care of each other” (hay que cuidarse entre si).

This deeply communitarian and highly improvisational approach to mitigating the coronavirus crisis is noteworthy for its emergence before large-scale formal responses by the political sector. At a moment when governments seemed unable to respond effectively and honestly to an unfolding crisis, *trovadores* and the online infrastructure that became their main expressive platform took on the role of trusted community voice.

## Conclusion

The COVID-19 pandemic and the social isolation that it produced across the world has posed an existential threat to various forms of cultural expression that, at their core, are oriented around group gatherings. During the spring of 2020, participatory media platforms constituted a means of maintaining connection during times of physical estrangement. In the transnational son huasteco community, the existing infrastructure developed by amateur archivists and DIY promoters to cater to the needs of a geographically dispersed cultural diaspora scaled to meet the needs of the community separated by the pandemic. Just as this digital community coalesced in an *ad hoc* and emergent fashion, so too did musicians respond to the coronavirus by composing new verses to address the crisis. Their new verses encouraged strategies for community preservation, sought to sooth uncertainty and fear with familiar repertory, and entertained listeners with humor in the face of isolation and nascent death. This study is an initial exploration of the linkages between tradition, innovation, digital platforms, and communities finding contingent ways of responding to the global coronavirus pandemic. This case study contributes to the growing body of literature on music and COVID-19 and highlights various dimensions of the ways localism and communitarianism were articulated across an increasingly interlinked transnational mediascape.

## Data Availability Statement

The original contributions presented in the study are included in the article/supplementary material, further inquiries can be directed to the corresponding author/s.

## Ethics Statement

Written informed consent was obtained from individuals interviewed for this study. Written informed consent was not obtained for the publication of any potentially identifiable images or data included in this article from individual(s) speaking in openly accessible online videos or online live chats.

## Author Contributions

All authors listed have made a substantial, direct and intellectual contribution to the work, and approved it for publication.

## Conflict of Interest

The authors declare that the research was conducted in the absence of any commercial or financial relationships that could be construed as a potential conflict of interest.

## Footnotes

^1^*Son Mexicano* can be a challenging concept to define. Son has been described as a “mega-genre” that integrates components of music (typically played on string instruments), verse (typically strophic), and choreography (often danced atop a *tarima* or *tabla*, a raised wooden platform) (Sturman, 2015, p. 105–106). Raquel Paraíso González defines *son* as “a generic term that describes the various regional traditions, each type associated with certain instrumental ensembles, dance styles, performance practice nuances, and texts, as well as the complex [structures] they form.” Paraíso notes that the concept of the *son* is intimately tied to the concept of *fandango*, the event at which *son* is enacted, which are currently understood as “events where all aspects of the Mexican son—dancing, singing, playing music, and poetry—are lived and experienced along with food, attire, language, verbal interactions, and a myriad of other cultural expressions” (González Paraíso, 2014, p. 446–8). Muñoz (2013, p. 48–50) notes that the terms *fandango* and *huapango* are used interchangeably by some practitioners, and that in the Huasteca region the term *huapango* is used to describe both the *sones* and the celebratory events at which it is performed. Significant scholarship has addressed the linkages between framings of identity and community manifest in various regional son traditions and revival movements (Sheehy, 1979; Bolaños, 2012). Additionally, many researchers have specifically theorized the symbolic poetics of son huasteco as marking and reaffirming shared ethnic, regional, or transnational identities (Hernández, 2000; Bonilla Burgos and Gómez Rojas, 2013). The literature on son huasteco in English is fairly limited. Related transnational aspects of Mexican son have received recent attention (Cardona and Rinaudo, 2017; Nieto, 2019).

^2^Utilizing the now familiar term pioneered by Appadurai (1996).

^3^This article specifically examines aspects of the digital communities surrounding música huasteca during the pandemic, but there are significant fruitful connections between this research and examinations of related “community music” making in the digital and virtual spaces Huasteca during the pandemic which will form the basis of future research projects. Here the authors are relying on theoretical work on community music making from English and Davidson (2020), Boeskov (2017), Krönig (2019), Matsunobu (2018), among others.

^4^All names of groups and individuals are taken from YouTube supplied titles and information as well as chat sessions and are presented following the established approaches to online ethnography in Sara Pink et al. (2016) and Patterson (2018). Spelling, capitalization, and textual idiosyncracies in quotes have been preserved. All interviewees provided written consent affirming the authors' permission to use their responses in this and future publications.

^5^Research on Facebook is made difficult by limited access issues (it is a closed platform to nonmembers) and, more significantly, because the search and filing system for the videos is not amenable to complex and multidimensional searching. Uploads can effectively disappear on Facebook in ways less common than on Youtube.

^6^Our use of the term communitarianism is drawn partially from the language of collaborative connectivity and membership, “needs fulfillment,” and sense of community described in the established model of McMillan and Chavis (1986) and reiteratedand broadened in Bermea et al. (2019).

^7^Huapango arribeño is a related but quite different style of traditional music from a region adjacent to the Huasteca. Son huasteco and huapango arribeño have a certain amount of overlapping appeal among many listeners, especially in the diaspora, despite differences in improvisational styles, instrumental technique, and dancing.
